# Association of Spontaneous and Induced Self-Affirmation With Smoking Cessation in Users of a Mobile App: Randomized Controlled Trial

**DOI:** 10.2196/18433

**Published:** 2021-03-05

**Authors:** Elizabeth L Seaman, Cendrine D Robinson, David Crane, Jennifer M Taber, Rebecca A Ferrer, Peter R Harris, William M P Klein

**Affiliations:** 1 CDC Foundation Atlanta, GA United States; 2 Behavioral Research Program (BRP) Division of Cancer Control and Population Sciences National Cancer Institute Rockville, MD United States; 3 Smoke Free 23 Ltd London United Kingdom; 4 Department of Psychological Sciences Kent State University Kent, OH United States; 5 School of Psychology University of Sussex Falmer, Brighton United Kingdom

**Keywords:** smoking cessation, smartphone, mHealth, sadness, self-affirmation, spontaneous self-affirmation, apps, mobile phone

## Abstract

**Background:**

Most smokers attempt to stop using cigarettes numerous times before successfully quitting. Cigarette cravings may undermine perceived competence to quit and thus constitute psychological threats to the individual’s self-concept. Self-affirmation may promote smoking cessation by offsetting these threats.

**Objective:**

This study examines whether self-affirmation is associated with smoking cessation in the context of a cessation app. Two types of self-affirmation are examined: tendency to spontaneously self-affirm, and self-affirmation inductions added to a publicly available smoking cessation app (Smoke-Free Quit Smoking Now). In addition, this study explores whether optimism and emotional states (happiness, anger, anxiousness, hopefulness, sadness) predict smoking cessation.

**Methods:**

All users who met the inclusion criteria, provided consent to participate, and completed a baseline assessment, including all individual difference measures, were randomized to 1 of 4 conditions. Half of the participants were randomly assigned to complete a self-affirmation induction upon study entry. Orthogonally, half of the participants were randomly assigned to receive self-affirming text notifications during their quit attempt or to receive conventional notifications. The induction and the text notifications were fully automated, and all data were collected through self-assessments in the app. Self-reported smoking cessation was assessed 1 month and 3 months following study entry.

**Results:**

The study enrolled 7899 participants; 647 completed the 1-month follow-up. Using an intent-to-treat analysis at the 1-month follow-up, 7.2% (569/7899) of participants self-reported not smoking in the previous week and 6.4% (503/7899) self-reported not smoking in the previous month. Greater tendency to spontaneously self-affirm predicted a greater likelihood of cessation (*P*<.001) at 1 month after controlling for smoking-related variables. Neither self-affirmation induction influenced cessation. In addition, spontaneous self-affirmation did not moderate the relationship between self-affirmation inductions and cessation. Greater baseline sadness was associated with a lower likelihood of reporting successful cessation. Optimism predicted past-week cessation at the 1-month follow-up, and both happiness and anger predicted past-month cessation at the 1-month follow-up; however, none of these potential predictors moderated the relationship between self-affirmation conditions and successful cessation.

**Conclusions:**

Spontaneous self-affirmation may be an important psychological resource for managing threats to self-concept during the smoking cessation process. Sadness may hinder quit attempts. Future research can explicate how spontaneous versus induced self-affirmation can promote smoking cessation and examine boundary conditions for the effectiveness of disseminated self-affirmation interventions.

**Trial Registration:**

ISRCTN Registry 56646695; https://www.isrctn.com/ISRCTN56646695

## Introduction

### Background

Tobacco use remains a leading cause of preventable death and disease globally, contributing to over 7.1 million deaths annually [1]. Each year, approximately 343,000 people in the United States die from cancer related to tobacco use [2]. Many adults are motivated to quit smoking cigarettes; however, most attempts to quit are unsuccessful [3,4]. Clinical practice guidelines emphasize combining pharmacological treatments with behavioral interventions [5]. There are several empirically supported behavioral treatments for smoking cessation [6]. However, the high rate of unsuccessful quit attempts [7] suggests that there is a need for supplementary and easily disseminable behavioral interventions.

### Mobile Health and Smoking Cessation

There is a growing body of literature supporting behavioral smoking cessation treatments delivered via mobile health platforms, including smartphones [8-10]. As smartphone access in the United States continues to rise, more individuals will have access to behavioral interventions delivered on smartphones. About 81% of US adults own a smartphone [11], and 72% of adult internet users have searched for health-related information on the web [12]. One international systematic review established that web-based health information seeking is common in many different countries and found that web-based health information seeking can improve patient-physician relationships [13].

Smartphone apps for smoking cessation can include a variety of theory-based intervention components that promote cessation, such as techniques to facilitate coping with craving and behavioral strategies for removing smoking-related stimuli from a smoker’s house [14]. Smoke Free-Quit Smoking Now is one such mobile app for smoking cessation that includes the behavior change techniques of supporting users to take on the identity of a nonsmoker, rewarding cessation, and changing routines [15].

### Self-Affirmation Theory and Applications to Smoking Cessation

Quitting smoking is a difficult endeavor, and most smokers attempt cessation many times before they successfully quit [16]. The process of attempting to quit smoking may in itself be psychologically threatening, as smokers may interpret cravings and temporary relapse during the process as indicators of lack of competence for quitting, constituting a threat to self-concept that results in negative affect [17]. When self-competence is threatened, it may undermine the cessation process by reducing motivation to quit or cessation self-efficacy. Many smoking cessation interventions bolster perceived competence to quit [18]. However, interventions to protect the self-concept are less common and may bolster the effectiveness of existing cessation interventions.

One such intervention approach is based on self-affirmation theory. According to this theory, people are highly motivated to see themselves as having *self-integrity*, which is marked by a sense of moral adequacy and competence [19]. Thus, when they experience threats to these attributes, they may respond defensively in an attempt to protect and bolster their self-integrity [20,21]. Health behavior change interventions often contain such threats because they suggest that one is volitionally engaged in a behavior that is harmful or irrational [22,23]. Defensiveness in the face of threats to self-integrity has been observed among smokers [24,25]. For example, smokers may respond to threatening cessation messages by impugning their content [26]. Even smoking cessation materials that are not explicitly threatening or loss-framed may be perceived as threatening by smokers attempting to quit or former smokers struggling with relapse. Self-affirmation theory suggests that to the extent that people can sustain views of themselves as morally adequate and competent, they will be more open to specific threats to the self. For example, smokers who are reassured about their self-integrity may be able to better face the challenges of cessation [27]. Accordingly, much research shows that when people have an opportunity to reflect on, for example, their cherished values before being exposed to threatening health information, such as a graphic warning label [24,28] or personal disease risk [29], they are more receptive to that information and may be more likely to engage in risk reduction behavior (meta-analyses [30-32]). We thus hypothesized that self-affirmation could offset the potential threats associated with quitting and, in turn, promote successful cessation.

Although evidence suggests that self-affirmation inductions can improve engagement with and efficacy of health behavior intentions, evidence is mixed for studies specifically targeting smokers. Some studies have found benefits of self-affirmation [28,33-38], including less defensiveness toward graphic warnings [28,36]. Moreover, when combined with other intervention strategies, such as motivational interviewing and cessation programs, self-affirmed individuals reduced cigarette consumption [37]. However, other studies have not found beneficial effects of self-affirmation on smoking-related outcomes for daily smokers [35,39,40]. Thus, additional research is needed to determine the effectiveness of induced self-affirmations among smokers.

In addition to research on self-affirmation inductions, some people are more likely than others to naturally or spontaneously engage in self-affirmation when feeling threatened or anxious [34,41-43]. Spontaneous self-affirmation may serve as a resource to facilitate smoking cessation because of its potential to offset cessation-related psychological threats in ways similar to induced self-affirmation. Indeed, the tendency to report spontaneous self-affirmation has been associated with greater acceptance of threatening health information [41,44], greater health information seeking [45], and other positive health-related outcomes, including higher perceived quality of care and increased likelihood of asking questions in a medical appointment [45-47].

There is some evidence that spontaneous self-affirmation may be beneficial for smokers. In one cross-sectional study of U.S. adult smokers, spontaneous self-affirmation moderated the relationship between living in a state with smoke-free policies, which may constitute a threatening environment for smokers, and quit intentions [48]. In this study, we examined whether spontaneous self-affirmation was associated with quit outcomes among a global cohort of smokers enrolled in a UK-based smoking app. In addition, we examined whether the tendency to spontaneously self-affirm moderated the relationship between self-affirmation inductions and successful cessation.

In addition to the tendency to spontaneously self-affirm, other psychological states and individual differences may serve as resources to bolster smoking cessation, either by interacting with self-affirmation or on their own. In this study, we examined optimism and sadness. Optimism refers to a general tendency to expect positive future events [49]. As optimism is a psychological resource that can bolster goal pursuit [50,51], people with higher optimism may have greater success at smoking cessation. Spontaneous self-affirmation and optimism are distinct psychological processes [41]; however, they may have similar associations with health outcomes, extending to smoking cessation [46]. Currently experienced emotions may also influence smoking cessation; such emotions may trigger action tendencies that facilitate predictable patterns of behavior [52-54]. Sadness, in particular, may be a hindrance to quitting smoking and predicting relapse during the smoking cessation process [55]. Sadness is associated with reward-seeking tendencies to mitigate loss [56], which can result in increased hedonically pleasing, but often unhealthy, appetitive behavior [57], including smoking [55]. Thus, when current or former smokers feel sad, they may turn to cigarettes in an attempt to improve their mood. In addition to influencing cessation success, emotion may influence the experience of relapse during the smoking cessation process.

### Self-Affirmation and Mobile Health: Creating Scalable Health Behavior Interventions

Health behavior interventions that are mobile or remotely delivered are easily implemented and widely disseminated, and integrating self-affirmation content could enhance their efficacy. The scalability of self-affirmation interventions has been demonstrated in other domains in which threat impedes adaptive outcomes (eg, education; [58]) but has rarely been examined in health contexts. Indeed, self-affirmation opportunities are disseminable, given that affirmation exercises require little time and effort but can have lasting effects [58,59]. Enduring effects from such a low-burden intervention are hypothesized to work through recursive processes [32]. That is, it is not necessarily the affirmation itself that continues to influence behavior over time. Rather, affirmation attenuates threats to self-competence that might arise from cravings and temporary relapse, which might otherwise impede motivation to quit smoking, and then allows individuals to capitalize on existing resources to facilitate behavior change [58,60]. A recent meta-analysis suggested that self-affirmation is more likely to facilitate change when psychological threat impedes behavior change and when resources are present to support such change [32]. Thus, implementing brief affirmations into an existing, scalable cessation intervention in which psychological threat may impede cessation may bolster the effectiveness of the intervention by maximizing the likelihood that individuals will benefit from the behavior change resources provided in the intervention.

### Previous Work Informing This Study

This study was designed as a follow-up to a previous study that provided initial evidence that incorporating a self-affirmation component into a standard text message–based smoking cessation intervention was a feasible, low-cost, and potentially efficacious way of bolstering the content of that intervention [61]. This previous study used a 2 (baseline affirmation: present vs absent) x 2 (integrated affirmation texts: present vs absent) factorial design [61]. In that study, 1261 participants met the eligibility criteria and initiated the program, 687 participants remained enrolled throughout the 42-day intervention, and 81 participants reported their smoking status at the end of the 42-day intervention [61]. Although there were no significant effects of affirmations on cessation when examining participants who remained enrolled in the study (n=687), affirmations did facilitate cessation when only participants who reported their smoking status at the 42-day follow-up were included in analyses (n=81, 6.4% of eligible baseline respondents) [61]. Intent-to-treat analyses of the 1261 participants who initiated the program indicated a 5.6% cessation prevalence at the 42-day follow-up [61]. This study builds on this former work by (1) providing a test of replication and (2) examining the role of individual differences in spontaneous self-affirmation, optimism, and affect.

### This Study and Hypotheses

This study was intended to replicate our team’s earlier study (Taber et al [61]) in a different setting. In this study, we tested whether self-affirmation was associated with better cessation outcomes in the context of a smoking cessation app. This study had 2 primary aims: to assess the effect of induced self-affirmation conditions on smoking cessation outcomes (aim 1) and to assess the associations of spontaneous self-affirmation with smoking cessation outcomes (aim 2). We hypothesized that 2 types of self-affirmation opportunities—a baseline kindness quiz and self-affirming push notifications in the subsequent months—would promote cessation. We also hypothesized that individuals with a tendency to spontaneously self-affirm at baseline would be more likely to successfully quit smoking. In the absence of relevant findings on which to base hypotheses, we tested whether induced self-affirmation conditions were more or less effective for people higher versus lower in spontaneous self-affirmation [41]. Finally, an exploratory aim (aim 3) was to assess baseline optimism and baseline affective states (happiness, anger, anxiousness, hopefulness, sadness) as potential predictors and potential moderators of the relationship between affirmation conditions and cessation outcomes.

## Methods

### Smoke Free App

Smoke Free-Quit Smoking Now is a UK-based app designed for iOS and Android devices [15] that attracts users from across the globe. The app averages 3000 new downloads a day and allows users to set a quit date and track their craving [15]. The app offers 4 methods by which users can monitor their progress: (1) a calendar showing the total amount of time since they stopped smoking, (2) a calculator that shows the amount of money saved by not buying cigarettes and the number of cigarettes not smoked, (3) virtual badges users can earn for milestones, such as 50 hours smoke-free, and (4) health progress indicators to monitor improvements since cessation [15].

### Participants, Recruitment, and Eligibility

A randomly selected proportion of users who downloaded the app during the study period (initially 10% and then increased to 30% to achieve recruitment goals) were shown a consent form and invited to participate in this study. In the informed consent form, participants were told that they could opt out of the study at any point by contacting the study investigator. The study employed a 2 (baseline self-affirmation induction: present vs absent) x 2 (notifications: self-affirming texts vs control texts) double-blind randomized controlled trial (RCT) in which self-affirmation opportunities were added to an existing smoking cessation app. Those who consented were randomly assigned to 1 of the 4 conditions and completed a baseline assessment. The initial recruitment goal was 5000 participants to have 500 completing the 1-month follow-up survey after accounting for 90% attrition, similar to previous studies [61,62]. A sample size of 500 at the 1-month follow-up was calculated to be able to detect a small effect size (*F*=.15), with high (.90) power using an analysis of variance (ANOVA) with 4 groups (calculated with G*Power). Participants were asked to complete 2 follow-up assessments 1 month and 3 months after they downloaded the app to assess cessation behavior and smoking status. All data were collected through the smartphone apps—users were notified about follow-up surveys in the app with one push notification and a red dot added to the app icon to indicate user action was requested. The link to complete the survey remained in the *Settings* section of the app until the participant responded.

**Figure 1:**
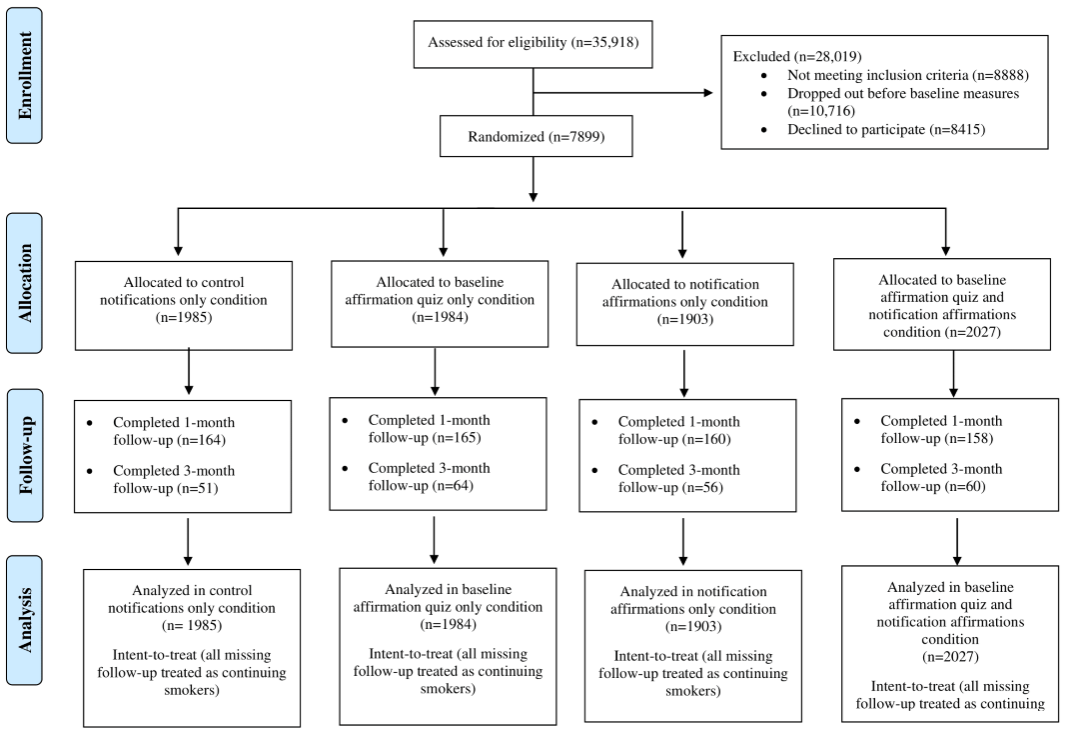
Consolidated Standards of Reporting Trials (CONSORT) diagram.

Once participants completed the baseline assessment, their eligibility was determined. Participants were not included in the study if they were under 18 years or over 98 years of age, selected a quit date more than 14 days in the future or more than 1 day in the past, paid for additional app features (Pro users), or did not complete the baseline assessment. In a divergence from the previous study [61], potential participants in this study were required to have listed a quit date after the day they downloaded the app; this ensured that participants randomized to the baseline affirmation condition would take the baseline affirmation quiz before attempting cessation. In addition, during data collection, a glitch occurred in which the same identifier was assigned to multiple participants; all users affected by this glitch were excluded from the study and are indicated in Figure 1 under the designation of *not meeting inclusion criteria*. App users who were not eligible for the study could still use the app. All participants were entered in a lottery—noncontingent on completion of surveys—for a US $100 Amazon gift card. This study was approved by the Institutional Review Board of the National Cancer Institute.

In total, 7899 participants met all inclusion criteria, were enrolled in the study, and provided survey responses. The country of participant residence was not assessed at the individual level; however, aggregate information about the geographic location of participants was available; most participants were from the United Kingdom, closely followed by the United States (Multimedia Appendix 1). Overall, the mean age of participants was 30.5 years (SD 8.7). The majority of participants were male (4790/7899, 60.6%) and did not use any cessation aids other than the Smoke Free app at baseline (6178/7899, 78.2%). Table 1 shows the demographic characteristics of participants at baseline. Significance assessed in Table 1 used a Bonferroni-corrected α level (.05 and 16 comparisons, so the adjusted α level is *P*<.003125).

**Table 1:** Baseline characteristics of the study participants (N=7899).

Characteristics		Control notifications only (n=1985)	Affirmation quiz only (n=1984)	Notification affirmations only (n=1903)	Both quiz and notification affirmations (n=2027)	Overall (N=7899)	Test statistics		*P* value^a^
							*F* test (*df*)	Chi-square (*df*)	
Age (years), mean (SD)		30.5 (8.7)	30.5 (8.6)	30.5 (8.8)	30.6 (8.6)	30.5 (8.7)	0.05 (7898)	N/A^b^	.99
**Gender, n (%)**							N/A	1.6 (3)	.65
	Male	1199 (60.4)	1225 (61.7)	1138 (59.8)	1228 (60.6)	4790 (60.6)			
	Female	786 (39.6)	759 (38.3)	765 (40.2)	799 (39.4)	3109 (39.4)			
**Currently using cessation aid, n (%)**							N/A	4.3 (3)	.23
	Yes	440 (22.2)	401 (20.2)	434 (22.8)	446 (22)	1721 (21.8)			
	No	1545 (77.8)	1583 (74)	1469 (77.2)	1581 (78)	6178 (78.2)			
**Time to first cigarette, nicotine dependence, n (%)**							N/A	5.4 (9)	.80
	Within 5 min	505 (25.4)	494 (24.9)	467 (24.5)	495 (24.4)	1961 (24.8)			
	6 to 30 min	386 (19.4)	369 (18.6)	361 (19)	411 (20.3)	1527 (19.3)			
	31 to 60 min	572 (28.8)	588 (29.6)	576 (30.3)	563 (27.8)	2299 (29.1)			
	After 60 min	520 (26.2)	533 (26.9)	499 (26.2)	558 (27.5)	2110 (26.7)			
	Missing^c^	2 (0.1)	0 (0)	0 (0)	0 (0)	2 (0.1)			
Desire to smoke^d^, mean (SD)		3.1 (1.4)	3.1 (1.4)	3.2 (1.3)	3.2 (1.3)	3.2 (1.3)	3.28 (7898)	N/A	.02
**Cessation stage of change, n (%)**							N/A	11.7 (3)	.009
	Yes, within next 30 days	1770 (89.2)	1897 (91.1)	1735 (91.2)	1795 (88.6)	7107 (90.0)			
	Yes, within the next 6 months or no	215 (10.8)	177 (8.9)	168 (8.8)	232 (11.4)	792 (10.0)			
Felt happy^e^, mean (SD)		3.2 (0.9)	3.3 (0.9)	3.2 (0.9)	3.2 (0.9)	3.2 (0.9)	0.73 (7898)	N/A	.54
Felt angry^e^, mean (SD)		2.9 (0.9)	2.8 (0.9)	2.9 (0.9)	2.8 (0.9)	2.8 (0.9)	0.50 (7898)	N/A	.68
Felt anxious^e^, mean (SD)		3.2 (1.1)	3.1 (1.1)	3.1 (1.1)	3.1 (1.1)	3.1 (1.1)	0.97 (7898)	N/A	.41
Felt hopeful^e^, mean (SD)		3.1 (1.0)	3.1 (1.0)	3.1 (1.0)	3.1 (1.0)	3.1 (1.0)	0.41 (7898)	N/A	.75
Felt sad^e^, mean (SD)		2.9 (1.0)	2.8 (1.0)	2.8 (1.0)	2.9 (1.0)	2.8 (1.0)	2.06 (7898)	N/A	.10
Tendency to spontaneously self-affirm^f^, mean (SD)		3.1 (1.1)	3.2 (1.1)	3.1 (1.2)	3.1 (1.1)	3.1 (1.1)	2.01 (7898)	N/A	.11
Optimism^f^, mean (SD)		3.6 (1.2)	3.7 (1.2)	3.6 (1.2)	3.6 (1.2)	3.6 (1.2)	2.28 (7898)	N/A	.08
Age started smoking^g^, mean (SD)		17.7 (4.2)	17.6 (4.1)	17.8 (3.9)	17.7 (4.3)	17.7 (4.1)	0.32 (7836)	N/A	.81
Cigarettes per day^h^, mean (SD)		14.7 (8.2)	14.6 (8.1)	14.7 (8.2)	14.7 (8.6)	14.7 (8.2)	0.14 (7882)	N/A	.94
Quit attempts in past year^i^, mean (SD)		7.9 (27.5)	6.5 (20.7)	6.6 (19.6)	7.2 (22.8)	7.9 (27.5)	1.55 (7884)	N/A	.20

^a^Significance assessed using the Bonferroni-corrected α level=0.05/16 comparisons=0.003125. No variables assessed met the threshold for statistical significance after the Bonferroni correction was applied.

^b^N/A: not applicable.

^c^Respondents who were missing a valid answer for time to first cigarette were included in all subsequent analyses if they had valid data for all other variables.

^d^1=not at all; 5=a lot.

^e^1=none of the time; 5=all of the time.

^f^1=strongly disagree; 5=strongly agree.

^g^Age started smoking was a write-in question; all ages from 5 years old to present age were considered valid cases. All ages outside of this range were excluded from analysis of age started smoking but were included in all subsequent analyses.

^h^Cigarettes per day was a write-in question; all values from 0 to 99 were considered valid cases. All responses outside of this range were excluded from the analysis of cigarettes per day but were included in all subsequent analyses.

^i^Past-year cessation attempts were assessed via a write-in question; all values from 0 to 365 were considered valid cases. All responses outside of this range were excluded from analysis of past year cessation attempts but were included in all subsequent analyses.

### Baseline Measures

Upon agreeing to participate, participants provided their age, gender, age at which they started smoking, average number of cigarettes smoked per day, smoking cessation aids (if any) they were currently using, and a quit date. Data on race and ethnicity were not collected, in part because the app is available globally and participants came from different countries with different racial and ethnic groups.

Participants were also asked questions to assess potential differences in baseline smoking behavior and levels of addiction. Baseline measures used to compare groups included nicotine dependence (*How soon after you wake up do you smoke your first cigarette?* [63]) and desire to smoke (*How strong is your desire to smoke, right now?* with options *not at all* to *a lot* on a 5-point scale). Previous quit attempts were assessed (*In the last year, how many times have you quit smoking for at least 24 hours?*). Smoking cessation stage of change was assessed using the following item: *Are you seriously thinking of quitting smoking?* with answer choices *yes, within the next 30 days*, corresponding to the Transtheoretical Model’s preparation stage, *yes, within the next 6 months*, representing the Transtheoretical Model’s contemplation stage, and *no, not thinking of quitting*, corresponding to precontemplation [64]. On the basis of the distribution of responses and our conceptual interest in the effects of self-affirmation among smokers who intend to quit smoking, the stage of change was dichotomized into *yes, within the next 30 days* and *yes, within the next 6 months* or *no*. These items were used to compare groups at baseline for smoking behavior and experiences.

Affect was assessed using items from the Positive and Negative Affect Schedule (PANAS) [65] as adapted by the Midlife in the United States (MIDUS) study [66,67] and the National Cancer Institute (NCI) Health Information National Trends Survey (HINTS). Participants rated their level of happiness, anger, anxiety, sadness, hopefulness, and anxiety within the past 30 days on a 5-point scale from *none of the time* to *all of the time*. The affect items were reverse coded so that higher scores indicated experiencing that emotion more often. Participants’ tendencies to engage in spontaneous self-affirmation were assessed as the average of 2 items used in previous studies [45,48] from the longer spontaneous self-affirmation measure (SSAM [41]): (1) “When I feel threatened or anxious I find myself thinking about my strengths” and (2) “When I feel threatened or anxious I find myself thinking about my values.” Participants’ baseline level of optimism was assessed with the item: “I’m always optimistic about my future” [68]. The SSAM and optimism items were assessed on a 5-point scale with the anchors 1=strongly agree to 5=strongly disagree. However, these items were reverse coded so that higher scores indicated more agreement and thus higher optimism.

### Follow-Up Surveys (1 and 3 Months)

All participants were invited to complete a 1-month and 3-month follow-up survey to assess smoking status. Both follow-up surveys assessed smoking status with the items: “Have you smoked at all in the past month?” and “Have you smoked at all in the past week?” Response options for both questions were “no, not a puff,” “1-5 cigarettes,” or “more than 5 cigarettes.” For these analyses, responses were dichotomized to indicate no smoking or smoking (1 cigarette or more) in the time period. In addition, participants were asked to report the average number of cigarettes smoked per day, time to first cigarette, and if they were using any other cessation aids at follow-up.

### Baseline Self-Affirmation Questionnaire

Participants assigned to the baseline affirmation conditions were shown a shortened, 5-item kindness questionnaire (quiz), adapted from previous work [61,69], directly after the baseline questionnaire. The purpose of this quiz was to induce self-affirmation by allowing participants to respond *yes* to having engaged in specific instances of past kindness. In the original questionnaire, participants were asked to provide written examples for each item to which they responded affirmatively; however, participants in this study were not asked to provide examples. This self-affirmation induction has been frequently used, has face validity, and is easy to implement [70]. The control condition did not receive the kindness quiz or any content in its place. The full self-affirmation questionnaire as well as responses by condition is presented in Multimedia Appendix 2. Of the respondents who received the baseline kindness quiz, approximately 83.1% (3333/4011) answered *yes* to 4 of the 5 items (Multimedia Appendix 2).

### Affirmation and Control Push Notifications

Participants in the control push notification condition received general tips related to quitting smoking, whereas participants in the affirmation push notifications condition received affirming messages from a pool of 15 possible notifications. The affirmation messages were based on literature and a previous study of self-affirmation content that had been integrated into a text messaging intervention for smoking cessation [61]. The control notifications were informed by the smoking cessation literature [71,72]. Participants received 2 notifications (either self-affirmation or control, depending on their assigned condition) per day for the duration of the study, unless they turned off notifications, which was the same as the frequency of notifications in the current version of the app. One notification was sent during each of the following time blocks: 8 AM to 2:30 PM and 2:31 PM to 9 PM. Participants were able to change the earliest and latest time for the notification (eg, change 8 AM to 7 AM or 9 PM to 11 PM). Participants could also access these self-affirmation or control messages (depending on condition) every time they reported experiencing a craving on a *Tips* screen. The full text of all notifications, organized by category, is presented in Multimedia Appendix 3.

### Analysis

All analyses were conducted using Stata 16 [73]. First, attrition rates were calculated for each of the 4 induced self-affirmation conditions. Second, demographic characteristics and baseline survey responses were compared across groups using ANOVA and chi-square tests. Third, a series of binary logistic regression models were run to examine predictors of successful cessation and potential moderating factors. All regression analyses used intent-to-treat, such that respondents who did not provide follow-up data were treated as continuing smokers. We elected to use intent-to-treat because it is widely used for assessing smoking cessation in interventions [8,9] and tends to be more conservative in assuming that all participants lost to follow-up continued to smoke instead of artificially inflating the cessation rate by removing participants lost to follow-up from analyses. We adopted *P*=.05 as our cut-off for statistical significance, with Bonferroni corrections applied for multiple comparisons as necessary.

### Trial Registration

The trial was retrospectively registered at ISRCTN: https://www.isrctn.com/ISRCTN56646695.

## Results

### Enrollment, Attrition, and Participant Characteristics

A CONSORT (Consolidated Standards of Reporting Trials) diagram is provided to show subject enrollment and study completion (Figure 1). Overall, 8.2% (647/7899) of users who enrolled in the study completed the 1-month follow-up survey and 2.9% (231/7899) completed the 3-month survey (Table 2), consistent with systematic reviews that found high levels of attrition in web-based RCTs [74]. The proportion of users who completed the follow-up was lower than in previous studies of this same app in which 7.5% of participants completed a 3-month follow-up [15]; however, the sample size in this study was considerably smaller. Attrition in this study was similar to attrition in a similar previous study in which 6.4% of participants completed a 42-day follow-up [61]. Although we estimated 90% attrition in our power calculations, we exceeded this percentage. In addition, there were more follow-up assessments in this study and it was available to Android but not iOS users, in contrast with the previous study [15], which had fewer and shorter follow-up assessments and enrolled both iOS and Android users. It is possible that the more frequent follow-ups combined with differences between the iOS and Android app can help contextualize this finding. Follow-up rates did not differ significantly by condition (1 month: *χ*^2^_3_=0.6, *P*=.90; 3 months: *χ*^2^_3_=1.5, *P*=.68; Table 2). This paper presents analyses for both the 1- and 3-month follow-ups; however, as the 3-month follow-up rates were considerably lower than the 1-month follow-up rates, most implications and conclusions focus on results from the 1-month follow-up.

**Table 2:** Attrition and cessation rates by study condition (N=7899).

Outcome		Control notifications only	Affirmation quiz only	Notification affirmations only	Both quiz and notification affirmations	Overall	Test statistic, chi square (*df*)	*P* value
Completed baseline survey, n		1985	1984	1903	2027	7899	—^a^	—
**Completed 1-month follow-up, n (%)**		164 (8.3)	165 (8.3)	160 (8.4)	158 (7.8)	647 (8.2)	0.6 (3)	.90
	Past week cessation at 1 month	145 (7.3)	143 (7.2)	139 (7.3)	142 (7.0)	569 (7.2)	0.2 (3)	.98
	Past month cessation at 1 month	133 (6.7)	128 (6.5)	128 (6.7)	114 (5.6)	503 (6.4)	2.7 (3)	.44
**Completed 3-month follow-up, n (%)**		51 (2.6)	64 (3.2)	56 (2.9)	60 (3.0)	231 (2.9)	1.5 (3)	.68
	Past week cessation at 3 months	48 (2.4)	58 (2.9)	55 (2.9)	54 (2.7)	215 (2.7)	1.2 (3)	.75
	Past month cessation at 3 months	38 (1.9)	49 (2.5)	48 (2.5)	46 (2.3)	181 (2.3)	2.0 (3)	.57

^a^No statistical tests were run.

### Smoking Cessation and Baseline Differences

Cessation rates did not differ significantly between groups at the 1-month (past-week cessation: *χ*^2^_3_=0.2, *P*=.98; past-month cessation: *χ*^2^_3_=2.7, *P*=.44; Table 2) or 3-month (past week cessation: *χ*^2^_3_=1.2, *P*=.75; past month cessation: *χ*^2^_3_=2.0, *P*=.57; Table 2) follow-up. Using an intent-to-treat analysis, the overall past-week cessation rate was 7.2% (569/7899) and the past-month cessation rate was 6.4% (503/7899) at the 1-month follow-up (Table 2). This is similar to the previous study of a text messaging program with affirmation content, which found 5.6% cessation at 6 weeks using intent-to-treat analysis [61]. Notably, despite randomization, participants differed in baseline desire to smoke (*F*_7898_=3.28; *P*=.02; Table 1) and cessation stage of change (*χ*^2^_3_=11.7; *P*=.009; Table 1) across conditions, although neither met the threshold for statistical significance after the Bonferroni correction was applied. The Bonferroni correction is conservative; because cessation stage of change differed at the *P*<.01 level and is likely related to the smoking cessation outcome, subsequent regression analyses controlled for baseline cessation stage of change.

### Aims 1 and 2: Self-Affirmation’s Associations With Cessation Outcomes

The primary aim of this study was to assess the impact of induced self-affirmation conditions on smoking cessation. The secondary aim of this study was to assess the associations of spontaneous self-affirmation with smoking cessation. To assess factors associated with cessation, binary logistic regression models were run using one of the 2 main outcomes (past-week cessation at 1 month and past-month cessation at 1 month) as the dependent variable. The 2 main 1-month outcomes were strongly correlated (*r*=0.93). In both regression models, tendency to spontaneously self-affirm at baseline was a significant predictor of cessation (Table 3), consistent with the hypotheses. However, neither self-affirmation study condition nor their interaction was significant in these models, indicating that providing opportunities for self-affirmation in the smoking cessation smartphone app did not result in a greater likelihood of cessation than using the smartphone app without affirmation.

**Table 3:** Primary self-affirmation regression models (N=7899).

Variable	Past-week cessation at 1 month			Past-month cessation at 1 month			
	OR^a^ (95% CI)	SE	*P* value		OR (95% CI)	SE	*P* value
Baseline affirmation	0.99 (0.78-1.26)	0.1	.92		0.96 (0.75-1.24)	0.1	.76
Notification affirmations	0.99 (0.77-1.26)	0.1	.92		0.99 (0.77-1.28)	0.1	.97
Baseline and notification affirmations interaction	0.98 (0.70-1.38)	0.2	.90		0.87 (0.60-1.12)	0.2	.44
Cessation stage of change: Yes, within the next 6 months or No^b^	0.80 (0.59-1.10)	0.1	.17		0.85 (0.62-1.18)	0.1	.40
Spontaneous self-affirmation	0.85 (0.79-0.92)	0.0	<.001^c^		0.90 (0.83-0.97)	0.0	.01^d^

^a^OR: odds ratio.

^b^Reference category: Yes, within the next 30 days.

^c^*P*<.001.

^d^*P*<.05.

In addition, despite low follow-up rates, both 3-month outcomes were also explored. There was a similarly high association between past-week and past-month cessation at the 3-month follow-up (*r*=0.91). None of the self-affirmation measures (tendency to spontaneously self-affirm, baseline affirmation condition, notification affirmation condition, the interaction of baseline, and notification affirmations) significantly predicted cessation in the main 3-month models (Multimedia Appendix 4).

Subsequently, we ran models testing a three-way interaction between our self-affirmation study conditions and spontaneous self-affirmation with both the 1-month and 3-month outcomes. This interaction was not significant, establishing that spontaneous self-affirmation did predict 1-month cessation in this study, but did not moderate the relationship between study conditions and cessation outcomes.

### Aim 3: Examining Potential Predictors and Moderators

The goal of exploratory aim 3 was to assess baseline optimism and baseline affective states as potential predictors and moderators of the relationship between affirmation conditions and cessation outcomes. To test this aim, we conducted regression analyses in which optimism and each of the 5 affective states (happiness, anger, anxiousness, hopefulness, sadness) were simultaneously added to the main regression models (Table 4). That is, we tested whether these factors predicted 1-week and 1-month cessation at 1 month when controlling for spontaneous self-affirmation, baseline affirmation condition, notification affirmation condition, their interaction, and cessation stage of change. In this model, tendency to spontaneously self-affirm still significantly predicted past-week cessation at the 1-month follow-up (*P*<.001) but did not significantly predict past-month cessation at the 1-month follow-up, although this association approached significance (*P*=.05). Lower sadness was a significant predictor of successful cessation at the 1-month follow-up for both past-week and past-month cessation (*P*=.002 and *P*=.007, respectively). Optimism predicted past-week cessation at the 1-month follow-up (*P*=.04) and both happiness and anger predicted past-month cessation at the 1-month follow-up (*P*=.03 for both). We also ran these with each potential predictor assessed separately and the results did not differ from the simultaneous model presented in Table 4.

**Table 4:** Regression models with potential predictors (N=7899).

Variable	Past-week cessation at 1 month				Past-month cessation at 1 month		
	OR^a^ (95% CI)	SE	*P* value	OR (95% CI)		SE	*P* value
Baseline affirmation	0.97 (0.77-1.24)	0.1	.83	0.95 (0.74-1.22)		0.1	.68
Notification affirmations	0.98 (0.77-1.25)	0.1	.87	0.99 (0.77-1.27)		0.1	.92
Baseline and notification affirmations interaction	1.00 (0.71-1.40)	0.2	.98	0.88 (0.61-1.26)		0.2	.48
Cessation stage of change: Yes, within the next 6 months or No^b^	0.81 (0.59-1.11)	0.1	.19	0.86 (0.63-1.19)		0.1	.37
Spontaneous self-affirmation	0.86 (0.79-0.94)	0.0	<.001^c^	0.91 (0.84-1.00)		0.0	.05
Optimism	0.92 (0.85-0.99)	0.0	.04^d^	0.93 (0.85-1.00)		0.0	.09
Happy	1.05 (0.93-1.18)	0.1	.42	0.91 (0.83-0.99)		0.0	.03^d^
Angry	0.90 (0.80-1.01)	0.1	.06	0.87 (0.78-0.99)		0.1	.03^d^
Anxious	0.97 (0.88-1.06)	0.1	.48	0.97 (0.88-1.08)		0.1	.61
Hopeful	1.04 (0.94-1.14)	0.1	.47	1.03 (0.93-1.14)		0.1	.56
Sad	0.84 (0.75-0.93)	0.1	.002^d^	0.85 (0.76-0.96)		0.1	.007^d^

^a^OR: odds ratio.

^b^Reference category: Yes, within the next 30 days.

^c^*P*<.001.

^d^*P*<.05.

Next, moderation analyses were conducted, with a three-way interaction term (baseline affirmation, notification affirmation, and potential moderator) for each of the affective states and optimism, run separately. No three-way interaction was significant. The full model for sadness is provided as an appendix (Multimedia Appendix 5); however, the models for other affective states and optimism have not been included because of space limitations.

We then conducted parallel analyses with the 3-month outcomes. None of the affective states were significant predictors of cessation in the past week or month at the 3-month follow-up; however, optimism was a predictor of cessation for both past-week and past-month cessation at the 3-month follow-up (*P=.*004 and *P*=.002, respectively; Multimedia Appendix 4). Due to low follow-up rates at the 3-month follow-up, potential moderation was not explored with the 3-month outcomes.

### Association of Spontaneous Self-Affirmation With Dependence

Previous work has found that smokers with a greater tendency to spontaneously self-affirm report more quit attempts and higher quit intentions, particularly when they live in states with more comprehensive smoke-free laws, highlighting factors that affect the cessation process [48]. Due to the significant effects of spontaneous self-affirmation in our study, we undertook additional analyses. The mean spontaneous self-affirmation scores in our study (mean 3.11 out of 5, SD 1.1) were comparable with those of past studies (mean 2.75 out of 4, SD 0.14 [46]; mean 3.12 out of 5, SD 0.86 [75]).

We examined whether spontaneous self-affirmation was associated with several dependence and quit intention measures in our sample to assess whether respondents who had a greater tendency to spontaneously self-affirm were less dependent on nicotine or had a stronger desire to quit at baseline, offering them an advantage. We computed correlations of spontaneous self-affirmation with the cessation stage of change, time to first cigarette, and quit intention. All were small (all correlation coefficient values were less than 0.1), indicating that smokers higher in tendency to self-affirm in this study were not necessarily less addicted or more intent to quit at baseline than smokers with lower tendencies to self-affirm.

## Discussion

### Principal Findings

In this study, opportunities for self-affirmation provided in the smartphone app (baseline self-affirmation quiz and self-affirmation notifications) did not significantly improve the likelihood of successful cessation. However, tendency to spontaneously self-affirm was a strong and significant predictor of cessation. Baseline sadness was associated with a lower likelihood of reporting successful cessation at the 1-month follow-up; optimism was significantly associated with past-week cessation at the 1-month follow-up, and happiness and anger were both significantly associated with past-month cessation at the 1-month follow-up. There were no interactions between any explored individual difference predictor and study conditions.

The spontaneous self-affirmation findings are consistent with previous findings that spontaneous self-affirmation was associated with improved psychological well-being and health care experiences [45,46], both of which may play a role in the smoking cessation process. In addition, although previous studies have found a relationship between spontaneous self-affirmation and quit attempts and intentions [48], we did not find a relationship between spontaneous self-affirmation and any dependence or quit measure in our sample. Thus, any association of spontaneous self-affirmation with quitting was not due to dependence or past quit attempts. Future work should explore this relationship in more detail to understand the specific benefit and ways to help smokers who do not tend to spontaneously self-affirm.

### Comparison With Previous Work

This study differs from previous smoking self-affirmation studies that typically provide participants with information about the negative health consequences of smoking. In this study, no explicitly threatening health information or loss-framed messages were provided, consistent with the positive focus of the app. The messages conveyed the benefits of quitting instead of the harms of smoking and were not hypothesized to constitute explicitly threatening health information. We did not directly assess whether participants perceived any of the material in the app to be threatening. One recent meta-analysis found that self-affirmation is less likely to facilitate change when psychological threat is minimal [32], which suggests that self-affirmation opportunities may have been less effective in this study in the absence of directly threatening information. Moreover, smokers may be aware of the health costs of smoking, and it is unknown to what extent information about the health consequences is novel to smokers, which could help to explain null self-affirmation effects in previous studies of smokers. These studies would benefit from pilot testing to determine whether threatening information about the health consequences of smoking is indeed perceived by smokers as threatening and novel. An alternative explanation is that our control messages were received well by respondents, offsetting our ability to observe any benefit of the self-affirmation messages.

Previous studies have reported mixed findings concerning whether self-affirmation inductions can assist smokers trying to quit; some studies have found benefits [28,30,33-35], whereas others have not [35,39,40,76]. Our finding that induced self-affirmations did not influence smoking behavior is consistent with multiple other studies that have shown null or even backfiring effects among smokers who undergo self-affirmation interventions [35]. It is possible that our baseline affirmation quiz and notification affirmations did not induce self-affirmation in participants; consistent with previous self-affirmation intervention studies, no manipulation check for affirmation was included, and it is difficult to assess whether participants were successfully affirmed. The original kindness quiz asks respondents to write down a specific time they engaged in the aforementioned action [69]. In this study, the baseline kindness quiz was adapted to ask participants to answer *yes* or *no* without explicitly asking them to write or think of an example, given that the affirmation intervention occurred through text messages.

It is a challenge to determine how best to adapt self-affirmation interventions developed in laboratory settings to the real world. In a previous study testing whether various adaptations of the kindness quiz differentially affected health cognitions and smoking intentions among a sample of online smokers, there were no significant differences depending on whether participants were asked to write examples, imagine examples, or were not asked to provide any examples [35]. However, that study also found that none of the self-affirmation conditions were more effective than the control conditions [35]. In addition, participants asked to provide written self-affirmation responses endorsed fewer affirmation questions than those not asked to provide written examples, suggesting that the writing was onerous [35]. In that study, for participants providing written examples, the intervention took nearly 7 times longer than it did for participants not asked to provide examples [35]. Thus, more research is needed to determine how to administer effective self-affirmation interventions to participants not in laboratory settings.

Our finding that additional opportunities for self-affirmation added to the smartphone app in this study did not have effects could be due to multiple factors. The self-affirmation content may not have been as noticeable as self-affirmations in other studies, given that participants did not complete the study in a more controlled laboratory setting. In addition, participants may have skimmed or otherwise not engaged with the self-affirmation content in this study. We also do not have data on the extent to which participants read or engage with induced self-affirmation materials. In addition, the existing app material was evidence-based and has already been found to be relatively effective on its own [15], thus identifying additional benefits of novel self-affirmation intervention material may have been difficult.

This study complements existing evidence concerning the distinctiveness of spontaneous self-affirmation from other psychological resources, such as optimism [41]. In this study, the single-item measure of optimism was only moderately correlated with spontaneous self-affirmation (*r*=0.46). Some previous work using a small number of items has found that spontaneous self-affirmation is related to greater optimism [46]. However, the correlation between the full measure of spontaneous self-affirmation and optimism is small (eg, *r*=0.22 as observed in a study by Harris et al [41]). Furthermore, cancer survivors who reported greater optimism reported better physical, mental, and cognitive health, even when controlling for spontaneous self-affirmation [77]. In this study, spontaneous self-affirmation was a significant predictor of 1-month cessation outcomes, whereas optimism was unrelated to 1-month cessation outcomes but predicted 3-month cessation outcomes. Optimism facilitates pursuit of goals [50,51], so it is noteworthy that it was not associated with smoking cessation goals at 1 month, whereas spontaneous self-affirmation did maintain such an association.

Interestingly, baseline sadness was significantly related to cessation outcomes at the 1-month follow-up. Feeling sadness less frequently at baseline was associated with a greater likelihood of reporting both past-week and past-month cessation. The relationship between sadness and cessation outcomes is consistent with previous theory and research suggesting that sadness facilitates reward-seeking tendencies that might undermine healthy behavior, including smoking cessation [55-57]. Optimism and all affective states (happiness, anger, anxiousness, hopefulness, sadness) were not found to moderate the relationship between the assigned affirmation conditions and successful cessation. Previous work has found that clinical diagnoses of anhedonia and depressed mood predict increased odds of relapse among smokers trying to quit [78]; however, this study is among the first to examine specific affective states and their association with successful cessation. Future work can further disentangle the relationship between sadness and cessation experiences.

### Limitations

This study has several limitations. As previously discussed, we do not have data concerning whether participants were successfully affirmed and to what extent they were engaged by the intervention. There are several methodological limitations. As data were collected from all users who downloaded the smartphone app, it was difficult to maintain strict experimental control. We were not able to monitor if or when participants turned off notifications, so we were unable to assess an individual’s exposure to the notification content. We were also unable to determine the geographic location of individual participants and were only able to access aggregate geographic information for the sample. Participants came largely from the United Kingdom and the United States, but there were participants from 8 other countries. In addition, smoking cessation is a complex process, and whereas many users who completed the baseline assessments did not complete follow-up assessments, we were not able to analytically determine why these participants discontinued responding and if they had deleted the smartphone app due to successful cessation or another reason. Another limitation is the use of 1 or 2-item measures of key constructs, such as spontaneous self-affirmation. However, these items have shown significant associations with outcomes in other studies [41], thus providing support for their validity. Similarly, we only assessed affect once at baseline. Emotions fluctuate over time, particularly during the difficult smoking cessation process. Future studies can monitor changes in affect during the process, such as with daily dairies, to better understand the role that affects plays in cessation. Finally, the attrition experienced in this study was higher than expected based on previous similar studies [15,61,74]. In this study, we found that 8.2% (647/7899) of users who enrolled in the study completed the 1-month follow-up survey and 2.9% (231/7899) completed the 3-month survey, which is lower than the 7.5% of participants who completed a 3-month follow-up during a previous trial of this same app [15]. In a previous study that informed the present study, 6.4% of participants completed a 42-day follow-up [61]. High attrition limits the interpretability of results such that it may have made it difficult to detect and reduce the generalizability of results, particularly at the 3-month follow-up. Furthermore, the intent-to-treat approach assumes that nonresponders are smokers, whereas it could be the case that nonresponders found the protocol burdensome.

However, these limitations are offset by several considerable strengths of this study. This study used a sample of real-life users, which allows for an assessment of how the app will function outside of a highly controlled laboratory setting. The study was also theoretically driven and provides preliminary evidence for the promise of spontaneous self-affirmation in smoking cessation. An additional strength of this study comes from the use of an already-existing, successful smoking cessation app with the addition of self-affirmation specific content.

### Conclusions

The results of this study provide evidence that spontaneous self-affirmation may be an important threat management psychological resource in the context of smoking cessation. They indicate the difficulties of creating effective self-affirmation inductions in smoking apps. There is a need to examine the effectiveness of smartphone app–delivered self-affirmations and to develop more effective affirmations in future dissemination work.

## Appendix

Multimedia Appendix 1 Geographic locations of the participants.

Multimedia Appendix 2 Baseline self-affirmation questionnaire and responses by condition.

Multimedia Appendix 3 Text notifications by study condition.

Multimedia Appendix 4 Regression models for 3-month outcomes.

Multimedia Appendix 5 Regression models to explore sadness as a potential moderator of self-affirmation conditions.

Multimedia Appendix 6 CONSORT-EHEALTH (V.1.6) checklist.

## Abbreviations

ANOVA: analysis of variance
OR: odds ratio
RCT: randomized controlled trial
SSAM: spontaneous self-affirmation measure
