# Supplementary figures and images for: Association of Spontaneous and Induced Self-Affirmation With Smoking Cessation in Users of a Mobile App: Randomized Controlled Trial
Source: J Med Internet Res. 2021 Mar 5;23(3):e18433. doi: 10.2196/18433 (PMC7980123; doi:10.2196/18433)

**Multimedia Appendix 1**. Geographic location of participants.


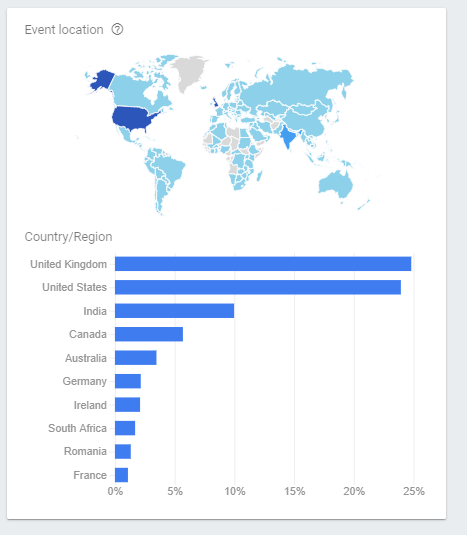

Supplement: Multimedia Appendix 1 [file jmir_v23i3e18433_app1.docx]
