# Supplementary material for: Association of Spontaneous and Induced Self-Affirmation With Smoking Cessation in Users of a Mobile App: Randomized Controlled Trial
Source: J Med Internet Res. 2021 Mar 5;23(3):e18433. doi: 10.2196/18433 (PMC7980123; doi:10.2196/18433)
Supplement: Multimedia Appendix 2 [file jmir_v23i3e18433_app2.docx]

**Multimedia Appendix 2**. Baseline self-affirmation questionnaire and responses by condition.

Tell us more about yourself

1. Have you ever been considerate of another person's feelings? If yes, spend a moment thinking of a specific time. Reply: Yes or No

2. Have you ever attended to the needs of another person? If yes, spend a moment thinking of a specific time. Reply: Yes or No

3. Have you ever forgiven another person when they have hurt you? If yes, spend a moment thinking of a specific time. Reply: Yes or No

4. Have you ever tried not to hurt the feelings of another person? If yes, spend a moment thinking of a specific time. Reply: Yes or No

5. Have you ever been generous and selfless to another person? If yes, spend a moment thinking of a specific time. Reply: Yes or No

|  |  | **Affirmation quiz only** | **Both affirmation quiz and notification affirmations** | |
| --- | --- | --- | --- | --- |
|  |  | **n=1984** | **n=2027** | |
|  |  | n (%) |  | n (%) |
| Answered “yes” to 0 items | | 63 (3.2%) | 76 (3.7%) | |
| Answered “yes” to 1 item | | 25 (1.3%) | 25 (1.2% | |
| Answered “yes” to 2 items | | 59 (3.0%) | 70 (3.5%) | |
| Answered “yes” to 3 items | | 179 (9.0%) | 199 (9.8%) | |
| Answered “yes” to 4 items | | 1,658 (83.6%) | 1,675 (81.7%) | |
| Answered “yes” to all 2 items | | 63 (3.2%) | 76 (3.7%) | |

Note: Participants were required to answer these questions “yes” or “no.” Any respondent who did not answer “yes” to a statement replied “no” (as opposed to skipping the item).
