# Supplementary material for: Association of Spontaneous and Induced Self-Affirmation With Smoking Cessation in Users of a Mobile App: Randomized Controlled Trial
Source: J Med Internet Res. 2021 Mar 5;23(3):e18433. doi: 10.2196/18433 (PMC7980123; doi:10.2196/18433)
Supplement: Multimedia Appendix 3 [file jmir_v23i3e18433_app3.docx]

**Multimedia Appendix 3.** Text notifications by study condition.

**Self-affirmation push notifications**

| **Tips to prevent relapse** |
| --- |
| When you feel like you might relapse, focus on your good experiences. Think about a time you made someone laugh. |
| When you feel like you might relapse, focus on your good experiences. Think about a time that you learned something new. |
| When you feel like you might relapse, think of a time you were honest with someone, even when it was hard. Reflect on that experience instead! |
| When you feel like you might relapse, think of a time you helped someone feel better about a bad situation. Reflect on that experience instead! |
| **Tips to stay motivated** |
| Quitting is hard! When you feel a craving, focus on a positive aspect of yourself. Think of a time you helped someone less fortunate than you are. |
| Quitting is hard! When you feel a craving, think of a time you learned from a mistake. |
| Quitting is hard! When you feel a craving, focus on what you stand for. Think of a time you treated people equally, regardless of who they were. |
| **Tips to manage cravings** |
| When you feel threatened by a craving to smoke, focus on your strengths. Think of a time you worked hard on something you care about. |
| When you feel threatened by a craving to smoke, focus on something important to you. Think of a time you helped a friend, even if you felt busy. |
| When you can’t take your mind off of smoking a cigarette, think of a time when you looked out for another person’s interests before your own. |
| When you can’t take your mind off of smoking a cigarette, think of a time when you tried to add some humor to something you were doing. |
| **Tips to manage your mood** |
| When you feel anxious about staying smokefree, think about a time you were loyal to someone you care about. |
| When you feel anxious about quitting smoking, focus on your values! Think of a time you showed compassion for another person, even if it was hard. |
| When you feel anxious about quitting smoking, focus on your values! Think of a time you stood up for what you believe in, even if it was hard. |
| When you feel anxious about staying smokefree, think about a time when someone else looked to you for advice. |

**Control push notifications**

| **Tips to prevent relapse** |
| --- |
| When you feel like you might relapse, remember the golden rule. Not another puff, no matter what. |
| When you feel like you might relapse, remember you’re saving money by not smoking. Spend the next few minutes thinking about all the money you saved. |
| When you feel like you might relapse, remember why you want to stop smoking? Bring some reasons to mind right now. |
| When you feel like you might relapse, picture your lungs filling with fresh, clean, air |
| **Tips to get active and stay motivated** |
| Get Active! Exercise, drink plenty of water, eat good food, get enough sleep |
| Get Active! take a walk, go for a run. Physical activity helps relieve stress, reduces cravings and gets you fitter. It also helps shed any excess weight you may have gained |
| Get Active! Short on time? No problem, even quick bursts of activity help. Walk up and down stairs a few times. |
| Get Active! If physical activity doesn’t feel right do some housework, file papers, call a friend |
| **Tips to manage cravings** |
| When you are craving, remember the four Ds: Delay, Drink water, Deep breathing, Do something |
| When you are craving, remember your mission is to get through the next 15 minutes without smoking |
| When you are craving, remember doing something will improve your mood, relieve stress and distract you from cravings. |
| If you’re having prolonged or severe cravings try nicotine replacement therapy |
| When you are craving, make a commitment to call someone before you light up |
| **Tips to distract yourself** |
| Distract Yourself! Have a book on hand on a subject you want to learn about. Read a few pages and make notes. You’ll be occupying your hands and your mind |
| Distract Yourself! Go online and read something, anything |
