# Supplementary material for: Association of Spontaneous and Induced Self-Affirmation With Smoking Cessation in Users of a Mobile App: Randomized Controlled Trial
Source: J Med Internet Res. 2021 Mar 5;23(3):e18433. doi: 10.2196/18433 (PMC7980123; doi:10.2196/18433)
Supplement: Multimedia Appendix 4 [file jmir_v23i3e18433_app4.docx]

**Multimedia Appendix 4:** Regression models for 3-month outcomes.

**Main self-affirmation regression models for 3-month follow-up**

|  | Past-week cessation  at 3 months | | |  | Past-month cessation  at 3 months | | |  |
| --- | --- | --- | --- | --- | --- | --- | --- | --- |
| Variable | OR(CI)^a^ | SE^b^ | *P* value |  | OR(CI)^a^ | SE^b^ | *P* value |  |
| Baseline affirmation | 1.20 (0.82,1.78) | 0.2 | 0.34 |  | 1.29 (0.84,1.98) | 0.3 | 0.25 |  |
| Notification affirmations | 1.19 (0.80,1.76) | 0.2 | 0.39 |  | 1.31 (0.85, 2.02) | 0.3 | 0.21 |  |
| Baseline and notification  affirmations interaction | 0.77 (0.45,1.33) | 0.2 | 0.35 |  | 0.71 (0.39,1.28) | 0.2 | 0.25 |  |
| Cessation stage of change (reference category: yes, within the next 30 days) |  |  |  |  |  |  |  |  |
| Yes, within the next 6 months or no | 0.58 (0.33,1.02) | 0.2 | 0.06 |  | 0.58 (0.31,1.07) | 0.2 | 0.08 |  |
| Spontaneous self-affirmation | 0.97 (0.86, 1.09) | 0.1 | 0.60 |  | 1.00 (0.88,1.13) | 0.1 | 0.95 |  |

^a^OR stands for Odds Ratio.

^b^SE stands for Standard Error.

**Regression models with potential predictors for 3-month follow-up**

|  | Past-week cessation  at 3 months | | |  | Past-month cessation  at 3 months | | |  |
| --- | --- | --- | --- | --- | --- | --- | --- | --- |
| Variable | OR(CI)^a^ | SE^b^ | *P* value |  | OR(CI)^a^ | SE^b^ | *P* value |  |
| Baseline affirmation | 1.19 (0.81, 1.76) | 0.2 | 0.37 |  | 1.27 (0.83,1.96) | 0.3 | 0.27 |  |
| Notification affirmations | 1.18 (0.79,1.74) | 0.2 | 0.42 |  | 1.30 (0.84,2.00) | 0.3 | 0.23 |  |
| Baseline and notification  affirmations interaction | 0.78 (0.45,1.34) | 0.2 | 0.37 |  | 0.71 (0.39,1.29) | 0.2 | 0.26 |  |
| Cessation stage of change (reference category: yes, within the next 30 days) |  |  |  |  |  |  |  |  |
| Yes, within the next 6 months or no | 0.58 (0.33,1.01) | 0.2 | 0.06 |  | 0.58 (0.31,1.07) | 0.2 | 0.08 |  |
| Spontaneous self-affirmation | 1.02 (0.89,1.17) | 0.1 | 0.78 |  | 1.06 (0.92,1.23) | 0.1 | 0.42 |  |
| Optimism | 0.83 (0.73,0.94) | 0.1 | 0.004^d^ |  | 0.80 (0.70,0.92) | 0.1 | 0.002^d^ |  |
| Happy | 1.04 (0.87,1.25) | 0.1 | 0.68 |  | 1.04 (0.85,1.26) | 0.1 | 0.73 |  |
| Angry | 0.90 (0.75,1.08) | 0.1 | 0.25 |  | 0.87 (0.72,1.06) | 0.1 | 0.18 |  |
| Anxious | 0.92 (0.79,1.07) | 0.1 | 0.27 |  | 0.92 (0.78,1.08) | 0.1 | 0.30 |  |
| Hopeful | 1.12 (0.96,1.31) | 0.1 | 0.16 |  | 1.14 (0.96,1.35) | 0.1 | 0.13 |  |
| Sad | 0.90 (0.76,1.08) | 0.1 | 0.25 |  | 0.90 (0.75,1.09) | 0.1 | 0.30 |  |

^a^OR stands for Odds Ratio.

^b^SE stands for Standard Error.

^c^ *P*<.05.

^d^*P*<.01.
