# Supplementary material for: Association of Spontaneous and Induced Self-Affirmation With Smoking Cessation in Users of a Mobile App: Randomized Controlled Trial
Source: J Med Internet Res. 2021 Mar 5;23(3):e18433. doi: 10.2196/18433 (PMC7980123; doi:10.2196/18433)
Supplement: Multimedia Appendix 5 [file jmir_v23i3e18433_app5.docx]

**Multimedia Appendix 5.** Regression models to explore sadness as a potential moderator of self-affirmation conditions.

|  | Past-week cessation  at 1 month | | |  | Past-month cessation  at 1 month | | |  |
| --- | --- | --- | --- | --- | --- | --- | --- | --- |
| Variable | OR(CI)^a^ | SE^b^ | *P* value |  | OR(CI)^a^ | SE^b^ | *P* value |  |
| Baseline affirmation | 1.24 (0.62,2.49) | 0.5 | 0.53 |  | 1.12 (0.54,2.32) | 0.4 | 0.75 |  |
| Notification affirmations | 0.96 (0.47,1.95) | 0.3 | 0.91 |  | 0.99 (0.47,2.06) | 0.4 | 0.97 |  |
| Baseline and notification  affirmations interaction | 0.88 (0.33,2.40) | 0.5 | 0.80 |  | 0.98 (0.34,2.83) | 0.4 | 0.97 |  |
| Sadness | 0.80 (0.68,0.96) | 0.1 | 0.01^c^ |  | 0.81 (0.68,0.97) | 0.1 | 0.02^c^ |  |
| Baseline and notification affirmations and sadness interaction | | | | | | | |  |
| No baseline affirmations, self-affirmation notifications | 1.00 (0.78,1.29) | 0.1 | 0.96 |  | 1.00 (0.77,1.29) | 0.1 | 0.99 |  |
| Baseline affirmations, control notifications | 0.91 (0.71,1.17) | 0.1 | 0.45 |  | 0.94 (0.72,1.21) | 0.1 | 0.62 |  |
| Baseline affirmations, self-affirmation notifications | 0.96 (0.75,1.23) | 0.1 | 0.76 |  | 0.90 (0.69,1.18) | 0.1 | 0.45 |  |
| Cessation stage of change (reference category: yes, within the next 30 days) |  |  |  |  |  |  |  |  |
| Yes, within the next 6 months or no | 0.81 (0.60,1.11) | 0.1 | 0.19 |  | 0.87 (0.63,1.20) | 0.1 | 0.39 |  |
| Spontaneous self-affirmation | 0.84 (0.78,0.90) | 0.0 | <0.001^e^ |  | 0.88 (0.82,0.96) | 0.0 | <0.001^e^ |  |

^a^OR stands for Odds Ratio.

^b^SE stands for Standard Error.

^c^ *P*<.05.

^d^*P*<.01.

^e^*P*<.001.
